# Why does the brain matter for education?

**DOI:** 10.1111/bjep.12727

**Published:** 2024-12-04

**Authors:** M. S. C. Thomas, Y. Arslan

**Affiliations:** ^1^ Centre for Educational Neuroscience University of London London UK; ^2^ School of Psychological Sciences Birkbeck, University of London London UK; ^3^ Department of Psychology and Human Development UCL Institute of Education London UK

**Keywords:** cognitive development, educational interventions, educational neuroscience, learning outcomes, mind, brain and education, teacher training

## Abstract

**Background:**

The present special issue on mind, brain and education (educational neuroscience) contains four papers that employ a neuroscience‐informed approach to educational phenomena, including dyslexia, academic self‐concepts, bullying and the effect of mindset on learning.

**Aim:**

This commentary positions the papers with respect to the goals and methods of educational neuroscience, placing them on a continuum of approaches from basic research to applied intervention.

**Procedure:**

We argue that a focus on the brain matters for teachers because it increases understanding of how learning works and the factors that influence learning outcomes and student well‐being without being reductionist. Constraints on learning that arise from biology sometimes seem arbitrary outside a neuroscience framework (several examples are provided). A neuroscience perspective encourages a more holistic and developmental view of learning than a narrow cognitive (memory) oriented approach. Because educational neuroscience is an inherently translational field that relies on dialogues between researchers and practitioners, we argue it is important to understand how teachers view the field and how insights from the science of learning might usefully feed into their practice. We then assess the insights, strengths and limitations of the four papers, as well as the potential that their respective lines of work offer.

## INTRODUCTION

This special issue of the British Journal of Educational Psychology focuses on *Mind, brain and education—Neuro‐mechanisms during child development*. It therefore invited contributions that focus on the contribution of neuroscience to the science of learning. The science of learning has been defined as the scientific study of the underlying bases of learning with the goal of describing, understanding or improving learning across developmental stages and diverse contexts (Privitera et al., 2023). The emphasis on neuroscience identifies the transdisciplinary field that seeks to translate evidence from biology, neuroscience and psychology into applications that will impact educational practices, where the term transdisciplinary intends a new field that is greater than the contribution of its component disciplines. The field has been given various names, including mind, brain and education, educational neuroscience, neuroeducation and neurodidactics. Here, we will use the term educational neuroscience.

This special issue contains four papers which cover, respectively, dyslexia (Åsberg Johnels et al., 2022), academic self‐concepts in adolescence (Rodriguez Buritica et al., 2024), bullying (Menabò et al., 2023) and the effect of mindset on learning (Janssen & van Atteveldt, 2022). In this commentary, we will consider which aspects of neuroscience influence these studies and where they sit on a continuum from basic to applied research, thereby placing them in a broader context. To set the scene, we first consider what is to be gained (and potentially lost) by introducing neuroscience into educational studies, and since the ultimate goal of this work is translation into classroom practice, what teachers currently make of educational neuroscience.

## THE RELEVANCE OF THE BRAIN FOR EDUCATORS

Why should the brain matter to educators? Psychology, with its hundred‐year body of research into learning and memory, provides plentiful usable research for teachers. It might not be immediately apparent why, say, the voltage potentials of populations of neurons as measured by scalp electrodes or the oxygenated blood flow to the prefrontal cortex as measured by a magnetic resonance imaging scanner should be relevant to how students learn in the classroom. Even if neuroscience data advance theories of learning in psychology, one could argue that it is the psychological theory that is relevant to educators rather than the details of biology. A focus on individual brains might also appear reductionist, obscuring the greater part of education that is about social entities like classes and schools, as well as decisions that societies must make about selecting curricula and how education systems are to be structured and funded.

One indication of why neuroscience is relevant to education can be found in artificial intelligence. We now know from research in computer science and robotics that there are many ways that cognitive systems can work in principle, many ways that information processing systems can learn and many ways that bodies can be controlled. The particular way that the human cognitive system works and the way that humans learn is due to the way their brains work. The way their brains work is due to biology. And our biology works the way it does because of evolution. The brain matters because teachers need to know how human cognitive systems work because of the foibles of biology. Indeed, if one views the mind as a form of information processing device, from the perspective of computer science, there are properties of learning in humans that seem strange until biology is considered (Thomas & Green, 2023).

For example, we find it difficult to learn very novel concepts that do not fit with our existing knowledge, so‐called arbitrary facts (van Kesteren et al., 2014), yet we find it easy to remember very novel events that happen to us (Frank & Kafkas, 2021; Lisman & Grace, 2005), particularly if the events are emotionally salient (Palombo et al., 2021). The reason is that these types of knowledge are stored in different brain systems (the cortex and hippocampus respectively), which operate by different principles and are modulated by neurotransmitters in different ways. The way we forget also differs according to the type of knowledge. We can easily forget rarely used facts, such as the name of the capital city of Mongolia (Davis & Zhong, 2017; Fisher & Radvansky, 2018), and facts need to be accessed to avoid forgetting them (MacLeod et al., 2018). But unless a therapist intervenes, we do not forget our phobias, such as a fear of spiders (Eaton et al., 2018; Herry et al., 2010; Kida, 2019; Milton, 2019). And even if we have not cycled for 10 years, when we climb on a bike, after the odd wobble, we will soon be riding competently (Nourrit‐Lucas et al., 2013; Romano et al., 2010). Again, the reason for the different types of forgetting is that different brain systems are involved (the cortex, the amygdala and the cerebellum), which operate by different principles. From a computer science perspective, there is no principled need for these types of knowledge to be stored in different systems, nor that they should have different forgetting curves, or indeed that knowledge should be forgotten at all. It is because of our biology that learning and forgetting work in these ways. This is the sort of information that is likely useful to teachers.

Moving beyond the cognitive, biology is also the reason that the brain keeps count of properties such as the metabolic costs of mental activity (Nagase et al., 2018). Thinking, especially when it involves focused attention, is costly in terms of energy consumption and the buildup of metabolic waste products. The brain has a budget and is continuously making decisions about whether continuing some current activity or launching into a new mental activity, is worth it. These computations involve assessing whether a task justifies the mental effort given the expected chances of success and the likely reward if successful (both influenced by prior experience) and given the alternative choices available in the moment which may offer other rewards (a product of context). Classroom learning frequently requires focused attention, which is why it can seem such hard work for children (and lead to messing around in the back row when not deemed worth it). In this case, knowledge of neuroscience allows teachers to understand the rationale behind some of the parameters within the classroom that will improve learning: limiting activities requiring focused attention, or interspersing them with rest; increasing student confidence in success; helping them understand the potential rewards of success; limiting opportunities for other distracting activities; and some contextual factors that may be important: how much thinking have the children done already, how tired are they already based on prior activities or the time of day, how fit are they?

### Neural mechanisms of learning

**FIGURE 1:**
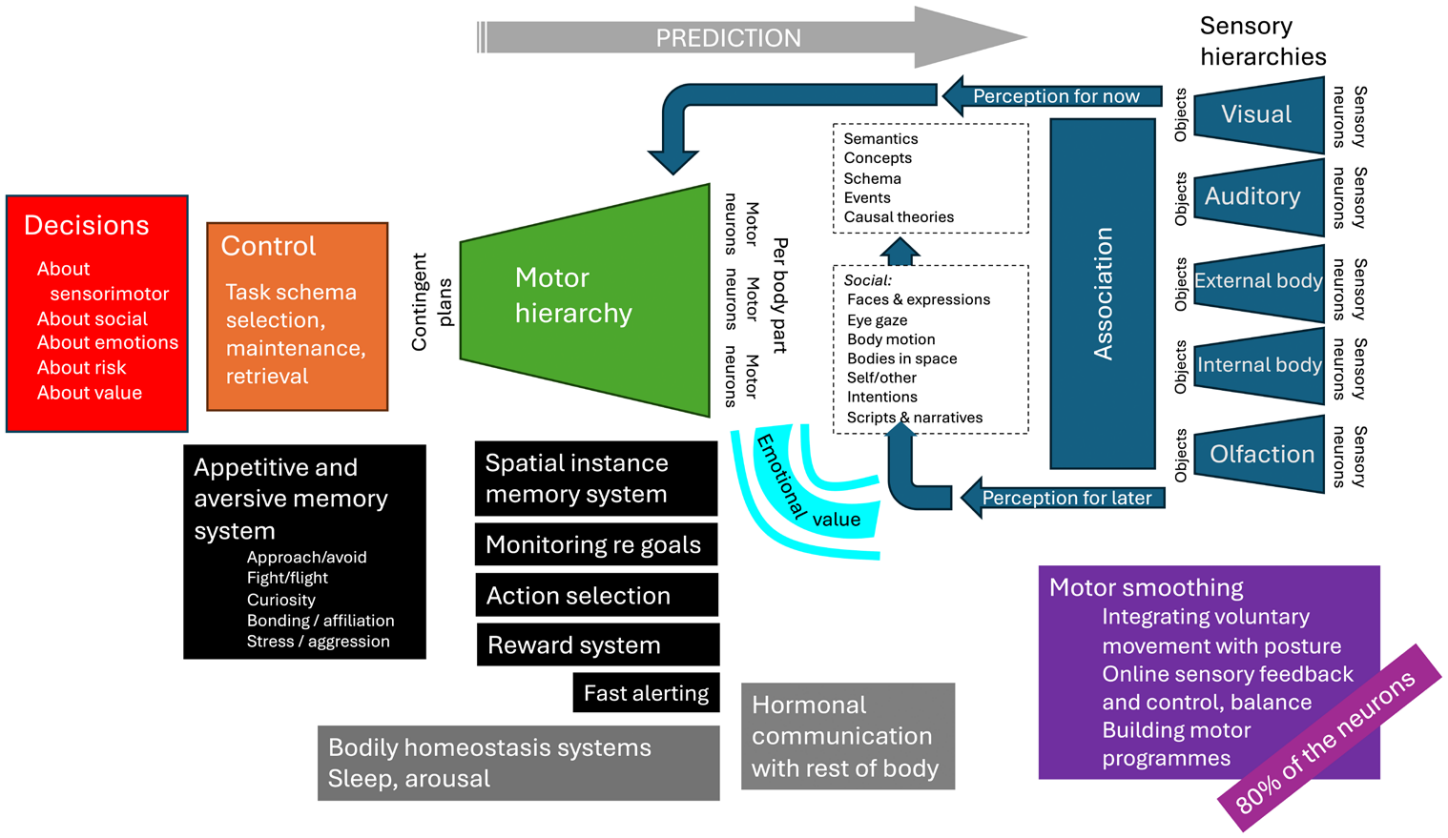
Broad functional architecture of the brain (Thomas & Green, 2023). Characterizing how the brain works in ways that constrain educationally relevant behaviour is a goal of educational neuroscience.

Neuroscience research has been building an understanding of the neural mechanisms of learning—and the factors that support or hinder them—since the 1990s, when the advent of new imaging technologies such as magnetic resonance imaging allowed researchers to explore *in vivo* brain functioning. The ultimate goal of this work is to characterize the brain's functional architecture and how this alters with development. We do not yet have a full picture because the brain is such a complex organ, but we know enough to sketch out what it will look like. Figure 1 shows the broad principles: hierarchical sensory systems and hierarchical motor systems interacting with emotion and reward systems and a significant proportion of resources dedicated to allowing the body to produce smooth, coordinated motor behaviour. Neuroanatomy is not key to this picture (though crucial for the research to reveal it). Still, particularly for translational goals, it is essential to consider how the brain differs from the types of information processing systems we have created in computer science, in order to understand the somewhat arbitrary ways in which humans learn and to tailor contexts to maximize learning outcomes and well‐being given these constraints.

For example, knowledge is stored in the strength of the connections between neurons in the brain. This means that it is hard to move around. This contrasts with a digital computer, where information is moved swiftly between working memory and long‐term memory. The consequence is that the brain uses systems dedicated to processing certain types of content and that content is built into each system's structure. The brain does not employ systems that can process any type of content, again in contrast to a digital computer's central processing unit (CPU). This is why cognitive training does not produce general benefits on thinking—because there are no general thinking systems to improve, only content‐specific systems which yield improvements on the particular task being trained. This has become clear in research that has investigated the training of one putative ‘general’ function, working memory, which does not yield general cognitive benefits (Nozari & Martin, 2024; Sala & Gobet, 2020). Therefore, if gains are task‐specific, to gain general benefits, students require training on a variety of tasks.

### The holistic view of learning encouraged by neuroscience

Neuroscience suggests that the brain is mainly a sensorimotor system, for integrating sensory information to generate movements to achieve goals. Emotions, motivations and rewards play key roles, and (as social primates) so do circuits oriented to other people. However, the brain also supports high‐level functions like complex reasoning and skills like literacy and numeracy. There are several ways to assess what the brain's relative processing priorities are: by indexing the length of evolutionary selection that has shaped relevant structures, assessed via the range of less closely related species across which structures and functions are observed; by the strength of genetic control on brain development, with tighter controls suggesting higher priority; and by the amount of neural real estate dedicated to different functions. By these measures, sensorimotor function is most important, followed by emotions and social processing. Cognition, including the sorts of knowledge and skills targeted by education, comes in fourth place. The educational implication, even at this coarse scale, is that teachers need to ensure the first three priorities—sensorimotor, emotional and social—at best enhance, and at worst do not impede, the fourth priority of cognition. A curious learner in a quiet classroom engaged in peer‐based learning will perform better than an anxious learner in a noisy classroom who does not want to appear too keen in front of their friends. A view of learning shaped by an understanding of how the brain works, therefore, leads to a more holistic perspective, stressing the importance of both the proximal sensorimotor environment and the socioemotional context (Duraiappah et al., 2021) beyond the narrower cognitive approach to instruction sometimes emphasized within psychology (e.g. Sweller, 2010).

### Pathways linking neuroscience and education

Several frameworks have been proposed for how neuroscience, psychology and education should interact in the transdisciplinary enterprise (e.g. Horvath & Donoghue, 2016; Stein & Fischer, 2011; Thomas et al., 2019). Here, we pick out three routes by which neuroscience and education can profitably interact, which are relevant to the four papers in the special issue. The first is the conventional route by which findings from neuroscience improve psychological theories of learning, the factors that influence learning, and individual differences in learning across typical and atypical populations: the interaction is *indirect* via psychology. In this pathway, the initial focus has been on developing a science of learning, but in its wake, a new science of teaching will develop, considering neural factors relevant to teachers' behaviour in the classroom. However, neuroscience may interact with education via a second *direct* route by virtue of the fact that the brain is a biological organ. There will be metabolic factors that influence its functioning, placing it in a better or worse state for learning. This route focuses on factors such as diet, exercise, sleep and stress. In a third route, neuroscience may interact with education by providing students, teachers and parents with self‐knowledge of how their brains work. This *indirect* route allows students to build meta‐cognitive strategies or reduce anxiety at the changes they may experience in transitional stages such as adolescence (e.g. Coleman, 2021).

### Types of study and methods used in educational neuroscience

To contextualize the four papers, it is useful to understand the different types of study that educational neuroscience employs. Some of them are still in effect basic research. Researchers are trying to discover how the brain works in the context of certain education‐relevant abilities, be it literacy, numeracy, creativity or social behaviour in the classroom, thereby revealing the constraints on learning that biology imposes. These insights might someday be of translational relevance. For our special issue, two of the papers are more of this flavour: the paper investigating the causes of dyslexia (Åsberg Johnels et al., 2022) and the paper investigating academic self‐concept in adolescence (Rodriguez Buritica et al., 2024). Some studies are more translational, producing findings that are a stepping stone towards developing new techniques or interventions, but fall short of proposing or evaluating an intervention. The paper by Menabò et al. (2023) on bullying is of this type, uncovering how children understand bullying situations, which is important for the development of future interventions. Other studies have moved into proposing specific educational interventions and evaluating their effectiveness, sometimes building towards full randomized controlled trials, where cognitive neuroscience underpins the theory of change. The paper by Janssen and van Atteveldt (2022) exemplifies this type of study in their evaluation of a growth mindset intervention.

Educational neuroscience also brings a set of tools for exploring learning. Rather than relying on accuracy and response times on behavioural tests, verbal reports, questionnaires or observation, neuroscientists deploy more sensitive measures which they hope will give new insights into underlying processes. These include measures of eye‐gaze, pupil dilation, skin conductance and heart rate variability, which are sensitive to emotional states and levels of stress, as well as cognitive processes such as attention. Multiple methods are used to triangulate brain processes, including measuring the electrical activity generated by neural firings (such as in electroencephalography [EEG] or magnetoencephalography [MEG]) or the energy consumption of different brain regions while performing a task (such as in functional magnetic resonance imaging [fMRI] or near infra‐red spectroscopy [fNIRs]). No method is ideal: some have better temporal resolution (accuracy in measuring the timing of brain activity), and some have better spatial resolution (accuracy in locating where brain activity occurs). Measures of brain structure, as assessed, for example, by MRI, are useful only for picking up changes over a long duration or for identifying the cumulative effect of environmental factors (such as poverty; see Farah, 2017). An overall picture emerges when multiple methods are used, combined with an understanding of neuroanatomy and low‐level function often gained from animal work.

### Potential benefits and risks of integrating neuroscience into education

Neuroscience will be useful to education if it helps teachers understand the factors that influence learning. That understanding need not involve detailed biology, neuroanatomy or mastery of neuroscience methods. For example, in Figure 1, we presented a ‘neuroanatomy‐free’ version of how the brain works to emphasize that the goal is understanding function, not relabelling or reduction. Perhaps, the greatest challenge for a translational field like educational neuroscience is that it needs to rely on a dialogue between researchers and practitioners to understand how insights from cognitive neuroscience can be implemented within the classroom to offer a benefit (and not just in principle but in the context of everyday classrooms—see next section); and for current classroom challenges to shape research agendas. Shortcomings in this dialogue and ways to improve it are the subject of current debates (e.g. Thomas et al., 2024), as are mappings between neuroscience insights and classroom practice in specific domains (e.g. see Bell & Thomas, 2024, for discussion in the domain of science teaching).

What of the charge that a focus on the brain is reductionist and overly narrow? This is perhaps an inevitable risk of focusing on brain function within the individual. However, researchers working in educational neuroscience have sought to stress the multiple nested levels at which factors may constrain learning and well‐being outcomes, from the individual to school, family, societal and governmental levels. Frameworks such as Bronfenbrenner's (1992) ecological systems theory and Michie et al.'s (2011) behaviour change wheel are used to emphasize that different levels beyond the individual child are relevant to educational outcomes and that these levels are interacting and co‐dependent (Thomas et al., 2019). Therefore, although neuroscience approaches involve studying individual brains, the transdisciplinary field of educational neuroscience views the individual level as only one constraint influencing learning outcomes and well‐being.

Nevertheless, there are potential pitfalls and distractions of integrating neuroscience into education. For example, neuroscience will not be useful for education if it only serves to relabel useful concepts from psychology with the names of neuroanatomical structures (such as replacing the term *executive function* with *prefrontal cortex* or *anxiety* with *amygdala*). It will not be useful if it merely spawns myths through poor communication, as found in so‐called neuromyths, such as the existence of ‘left‐brain thinkers’ and ‘right‐brain thinkers’ (e.g. Grospietsch & Lins, 2021). It will not be useful if it is used merely as neuroscience window‐dressing to make certain educational techniques seem more believable (a contextual framing effect taken from advertising, but here, often referred to as the ‘seductive allure of neuroscience’; Weisberg et al., 2008). And it will not be useful if it encourages a reductionist urge in science to explain all phenomena at the lowest level of description. Neural activity per se, such as that revealed in brain scans, will rarely be directly relevant to the classroom, and as argued by Horvath and Donoghue (2016), evidence from the neural level must be behaviourally translated before any prescriptive educational applicability can be proposed.

## TEACHERS' VIEWS ON EDUCATIONAL NEUROSCIENCE

Educational neuroscience exists to be translational. Therefore, it is important to understand the context of the education system into which it integrates. What do teachers think of the field and its potential to influence their pedagogy? What key questions do they have for researchers? How is knowledge of the brain currently incorporated into teacher training, and what barriers exist for current teachers to access this knowledge? What support is available to help teachers implement research insights in the classroom? We consider these questions in this section in the context of the United Kingdom.

The initial teacher training (ITT) programmes in the UK equip teachers with essential skills and knowledge for effective teaching in schools. These programmes cover key areas such as understanding the curriculum and subject knowledge, lesson planning and teaching methods, assessment and feedback, behaviour management and special educational needs (SEN) training (Department for Education, 2019b).

In the current content, there is growing recognition that, in addition to these foundational elements, knowledge of brain systems and learning processes may contribute to enhancing teaching practices (Arslan et al., 2022; Brick et al., 2021; Dubinsky et al., 2019; Privitera, 2021; Walker et al., 2019). This may be especially the case for those working with children with SEN, as it provides deeper insights into their unique needs (Howard‐Jones et al., 2020; Thomas et al., 2019). This is reflected in a growing interest in educational neuroscience among educators (Im et al., 2018; Pickering & Howard‐Jones, 2007; Wilcox et al., 2021), but this interest does not come without reservations. We consider the teachers' perspective at two levels, from focus groups run at the school level and from a national survey.

### Teachers' perceptions of the value of educational neuroscience

At the school level, a recent focus group study (Arslan et al., 2024) explored UK teachers' perceptions of the value of educational neuroscience in the teaching profession and identified barriers to accessing such training and materials. Participants, including primary and secondary school teachers, SEN specialists and school leaders, had varying levels of exposure to educational neuroscience, from continuing professional development (CPD) courses to informal resources like blogs and magazines.

When exploring the challenges surrounding CPD programmes related to educational neuroscience, teachers articulated many obstacles impeding their access to relevant training and resources. The scarcity of CPD programmes tailored to educational neuroscience emerged as a prominent concern among teachers. The data showed that time constraints were a common issue, with participants feeling frustrated about not having enough time for professional development. One participant captured this feeling by saying, ‘There's limited time for anything’, highlighting teachers' difficulty finding time for educational‐neuroscience‐focused CPD among their many duties.

Financial constraints also loom large as a significant barrier, as indicated by teachers' references to the costs associated with training programmes. The notion of being ‘constrained to a certain extent by the cost involved’ highlights the financial burdens placed on both individual teachers and schools in pursuing educational‐neuroscience‐focused CPD initiatives. Moreover, prioritizing other training initiatives over educational‐neuroscience‐focused CPD further compounds the issue. Participants noted that educational neuroscience training might not be perceived as a priority within educational institutions, with other areas, such as curriculum development or administrative training, taking precedence.

Substantial barriers to integrating educational neuroscience content were highlighted within ITT programmes. Participants pointed out a lack of emphasis on neuroscience in ITT curricula, noting that their training did not comprehensively cover this topic. This highlights the inadequacies of ITT in promoting a nuanced understanding of educational neuroscience among prospective teachers. ITT programmes tended to prioritize curriculum content over broader pedagogical principles, including educational neuroscience. Participants criticized the limited focus on child development, social–emotional learning and neuroscience, which are crucial for understanding effective teaching practices. The data revealed a disconnect between the theoretical foundations of ITT programmes and the practical realities of teaching. Teachers observed a gap between the teaching strategies taught during training and the challenges faced in real‐world classrooms.

The results showed a strong interest in integrating educational neuroscience into teaching practices, particularly in understanding cognitive mechanisms and the transformative potential of educational neuroscience knowledge in addressing individual learning needs and promoting inclusive education. Participants in the teacher focus groups acknowledged that cognitive mechanisms differ among individuals, especially those with SEN, which necessitates a nuanced understanding and application of educational neuroscience principles with individualized pedagogical strategies. Participants also noted that while they often apply neuroscientific principles unconsciously, formal training could enhance their understanding and application of these concepts. One teacher remarked, ‘Teachers know more than they consciously think they do… if you start to pause and think about it, you probably do know a lot more about it as a teacher, or you do put it into effect and think about it but without necessarily using the terminology’. This highlights the implicit use of educational neuroscience concepts and the need for explicit training to fully leverage their potential.

Teachers expressed a desire for enhanced educational neuroscience training opportunities, advocating for comprehensive programmes that promote deeper understanding and practical application. While recognizing the importance of educational neuroscience, participants nevertheless emphasized the need for accessible and engaging training formats that cater to diverse learning preferences and professional backgrounds. Furthermore, teachers stressed the importance of understanding how educational neuroscience can directly impact classroom practices and student outcomes. One participant asked, ‘How is this going to make a practical difference to how I help my pupils learn?’, highlighting the necessity for educational neuroscience to offer concrete benefits in enhancing teaching effectiveness and student achievement. This reflects the importance of offering practical guidance and resources to support teachers in effectively applying educational neuroscience concepts in their classrooms.

### Results of a national survey

Consistent with this small‐scale, in‐depth study, a recent representative national survey of over 1000 UK teachers assessed views of educational neuroscience and found that teachers favoured incorporating it into teaching practices (see Thomas et al., 2024; YouGov, 2022). A significant majority were aware of its benefits, such as understanding brain development stages and addressing individual learning difficulties. Additionally, 71% of teachers agreed that educational neuroscience is relevant to their professional development, and 55% believed implementing these insights in their classrooms was feasible if provided with practical guidance. The study also pointed out that many teachers are already familiar with educational neuroscience concepts, often through informal channels like online resources, but they lacked structured training that could enhance their application of these principles in the classroom. Nevertheless, when teachers were asked what concerned them most about their current practice, pedagogy was not to the fore. Teachers identified workload as the most significant issue they were facing (42%), followed by funding cuts (19%) and student behaviour (13%). This is the context into which any enquiry about pedagogical approaches and the potential contribution of educational neuroscience falls.

### Implications for teacher training and CPD

Together, the findings of school‐level and national studies underscore the growing interest in integrating educational neuroscience into teaching practices and the necessity for structured and systematic approaches to embedding educational neuroscience in ITT programmes. Teachers are willing to embrace these insights, provided they receive adequate training and resources. Despite the enthusiasm, there is a notable gap in formal training opportunities that offer practical applications of neuroscience theories. Currently, while ITT programmes cover many critical aspects for effective teaching, there is no formal requirement for teachers to train in educational neuroscience (Blanchette Sarrasin et al., 2019; Privitera, 2021), and most ITT programmes lack a comprehensive focus on the neurocognitive mechanisms of learning (Arslan et al., 2022; McMahon et al., 2019; Thomas et al., 2024; Tokuhama‐Espinosa & Nouri, 2023).

At the governmental level, the UK Department for Education sets frameworks and content for ITT programmes. The Early Career Framework (ECF) (Department for Education, 2019a) and Initial Teacher Training Core Content Framework (CCF) (Department for Education, 2019b) were introduced to establish teacher standards and enhance knowledge. The ECF specified what early career teachers should learn and practice as they begin their careers, while the CCF detailed the minimum content all trainee teachers should receive based on the best available evidence. It outlined the essential content that ITT providers and their partnerships must incorporate when designing and delivering their ITT programmes. In January 2024, the Department published the Initial Teacher Training and Early Career Framework (ITTECF), combining and refining previous frameworks to better address contemporary educational needs (Department for Education, 2024), aiming to provide a structured approach to developing the core pedagogical knowledge and skills necessary for teachers.

The ITTECF includes updates on supporting pupils with SEN, high‐quality oral language (oracy) and early cognitive development. A new statement on evidence literacy has also been added, highlighting the importance of teachers' engagement with evidence. It covers a broad range of topics designed to prepare new teachers effectively. While the framework includes cognitive elements of the science of learning, such as memory and attention, the crowded ITT curriculum often limits the depth of coverage and details of application. These concepts are introduced primarily during early career induction once teachers are in practice rather than as foundational knowledge during their initial training. For example, the framework's section on ‘How Pupils Learn’ emphasizes understanding working memory, long‐term memory and processes that enhance memory retention and learning outcomes (p. 13–14), but the practical application and deeper integration of these concepts are left for development during the early career period.

The current omission of educational neuroscience content knowledge has two consequences. First, it leads to a narrow focus on cognitive aspects of learning. It is not holistic, omitting socioemotional elements, and it is not developmental, for example, omitting important changes in executive functioning skills across mid‐childhood and the range of changes that accompany adolescence. Second, teachers with a thirst for neuroscience knowledge, sensing its importance, end up accessing such knowledge through informal and unstructured channels (Pickering & Howard‐Jones, 2007). Most of these aim at creating a one‐way translation of neuroscientific knowledge to classroom practice (Privitera, 2021) rather than fostering a reciprocal exchange between researchers and practitioners. In some instances, this can result in teachers developing specialized teaching skills via trial and error (Oliver et al., 2018).

When teachers encounter educational neuroscience content during their careers through informal paths, they face challenges in engaging with relevant research evidence and applying neuroscience concepts correctly for various reasons (Schwartz et al., 2019; Tan & Amiel, 2022). These reasons include a lack of previous exposure to neuroscience, leading to difficulties in grasping complex terminology and concepts (Schwartz et al., 2019); the persistence of neuromyths and misconceptions in educational settings (Tan & Amiel, 2022; Torrijos‐Muelas et al., 2021); and insufficient professional development opportunities that provide practical applications of neuroscience theories to classroom instruction (Tan & Amiel, 2022; Thomas et al., 2024). Additionally, the inherent complexity of translating scientific findings into educational practices complicates teachers' ability to effectively apply neuroscience concepts (Schwartz et al., 2019), which can result in ineffective teaching methods (Tardif et al., 2015) and susceptibility to embracing neuromyths (Arslan et al., 2022; Dekker et al., 2012; Gini et al., 2021; Privitera, 2021; Torrijos‐Muelas et al., 2021).

In sum, educational neuroscience falls into a UK context of enthusiastic teachers who, however, have limited time and opportunity to engage with a neuroscience‐informed pedagogical approach. This challenge could be addressed by a comprehensive introduction to foundational knowledge and principles in educational neuroscience during the initial stages of teacher training to ensure that teachers enter the classroom with a robust understanding of these concepts.

## THE PAPERS IN THE SPECIAL ISSUE

Now that we have characterized the goal of introducing neuroscience findings and methods to educational studies, and outlined how educational neuroscience interfaces (or not) with teacher training, we can situate the four papers. We will do so with respect to the methods they use and where they are placed on a continuum from basic to applied research.

### Åsberg Johnels et al.

Åsberg Johnels et al.'s paper entitled ‘Left visual field bias during face perception aligns with individual differences in reading skills and is absent in dyslexia’ (Åsberg Johnels et al., 2022) lies at the basic end of the basic‐applied continuum. Its goal is to shed light on the limiting factors impairing reading development in some children. Its neuroscience basis is the differential involvement of the brain's two hemispheres in processing visual stimuli. The study employs the sensitive measure of eye gaze to probe the relationship between two abilities: face recognition and reading. A key dimension in this paper is laterality—which side of the brain plays a greater role in the recognition of faces. The paper builds on an observation from cognitive neuroscience that face recognition that is more lateralized to the right hemisphere tends to be associated with greater attention to the left side of faces. From an educational perspective, it is not immediately obvious how face recognition and brain laterality are relevant for reading development, which makes the paper intriguing.

Forty‐six children aged 9–13, including 17 with dyslexia, were shown an 11‐second video of a female face. The children without dyslexia tended to look more at the left‐hand side of the face, and for these children, the more they tended to look at the left side of the face, the better were their reading scores. The dyslexics showed no overall bias for looking at the left‐hand side of the face, although we cannot tell whether their reading ability was related to a left‐side viewing preference due to floor effects in the reading measure. We have here evidence that face recognition works differently in the dyslexic group, perhaps suggesting that face processing is less lateralized to the right hemisphere and that in the non‐dyslexic group, there is a relationship between reading ability and how faces are viewed.

What does this mean? We have written elsewhere that, on the whole, which side of the brain is involved in cognition tends not to play an important role in behaviour (Thomas & Green, 2023). Functionally and structurally, the two sides of the brain tend to be mirror images of each other, with equivalent areas doing largely similar things, and the two sides tending to work together in most tasks. Some skills show greater lateralization, language being the most notable so that in adults, loss of language (aphasia) tends to be associated with damage to the left hemisphere. What is crucial here is that this hemispheric specialization is the *outcome* of a developmental process (Mareschal et al., 2007). In infancy, the two hemispheres have similar functions but are tuned slightly differently; they compete to take on tasks and then specialize; the left hemisphere is better tuned for the structural components of language and becomes dominant across development, while the right hemisphere takes on the processing of the melodic, emotional and contextual elements of language which are more holistic in nature. Reading is linked to this pattern of emergent specialization. Initially, face recognition in 5‐ to 6‐year‐olds is handled by the visual systems in both hemispheres. When a child's brain is required to learn expertise in associating visual patterns with spoken words, the efficient hemisphere to develop this skill in is the left, where oral language (particularly phonology and vocabulary) is processed, but this means the right hemisphere must take on a greater role in face recognition (see Liu et al., 2024, for recent work). Johnels et al.'s finding shows two things: that this developmental process of hemispheric specialization for faces is disrupted in dyslexics, and that in typical readers, greater specialization to the right hemisphere for face processing is linked with better reading. Hemispheric specialization, then, appears to be a marker of things going well developmentally.

What does it mean for teachers? Here, the authors are less confident, indicating they are agnostic about the developmental dynamics. By examining face recognition, they have identified a marker of a different trajectory of development, a trajectory that works less well for reading. However, further details of the atypical mechanisms are required for insights that might lead to potential methods of instruction for those struggling to learn to read. The long‐term goal is for neuroscience to contribute to education by improving psychological theories of learning to read, but here, the research still represents an exploration of basic mechanisms of development.

### Rodriguez Buritica et al.

Rodriguez Buritica et al.'s paper ‘Neural and behavioural correlates of adolescents' changing academic self‐concept’ (Rodriguez Buritica et al., 2024) is also mainly basic science, tracking changes in academic self‐concept in 13‐year‐olds across 18 months when students' views of their own abilities often decline. The paper presents two perspectives. One is behavioural, reporting the observation that adolescents' perception of their academic abilities/performance is associated with their actual academic achievement and with positive teacher evaluations. The authors argue that academic self‐concept may promote academic achievement, so ways to enhance self‐concept may have beneficial outcomes. Higher achievement and positive teacher feedback can help to improve academic self‐concept.

The second perspective is the neural one. Thirty‐nine children were placed in an MRI scanner and asked to decide whether or not a given trait adjective (e.g. naïve, smart) described themselves, their friends, or their teachers. Functional MRI measures the regions of the brain that are consuming more energy (demanding more oxygenated blood) during task performance compared to a baseline. Here, the regions turned out to depend on the condition that the children were performing, whether they were making the evaluations about themselves or friends or teachers. The same pattern was seen at the beginning and end of the 18 months, showing that developmental changes can be gradual. The authors reported that the modulatory effect of the condition was observed across a widespread network of cortical midline structures as well as frontal, temporal and parietal regions, including the ventromedial prefrontal cortex, anterior cingulate cortex, anterior insula, superior temporal gyrus, precuneus and lingual gyrus. When the authors used fMRI measures to predict changes in academic self‐concept, there were no clear findings (there were some interaction effects, but these cannot be taken as robust). The study illustrates the challenge of directly linking brain activations and a complex psychological construct like academic self‐concept. Without a correlation, the imaging measures need to be considered on their own merit.

**FIGURE 2:**
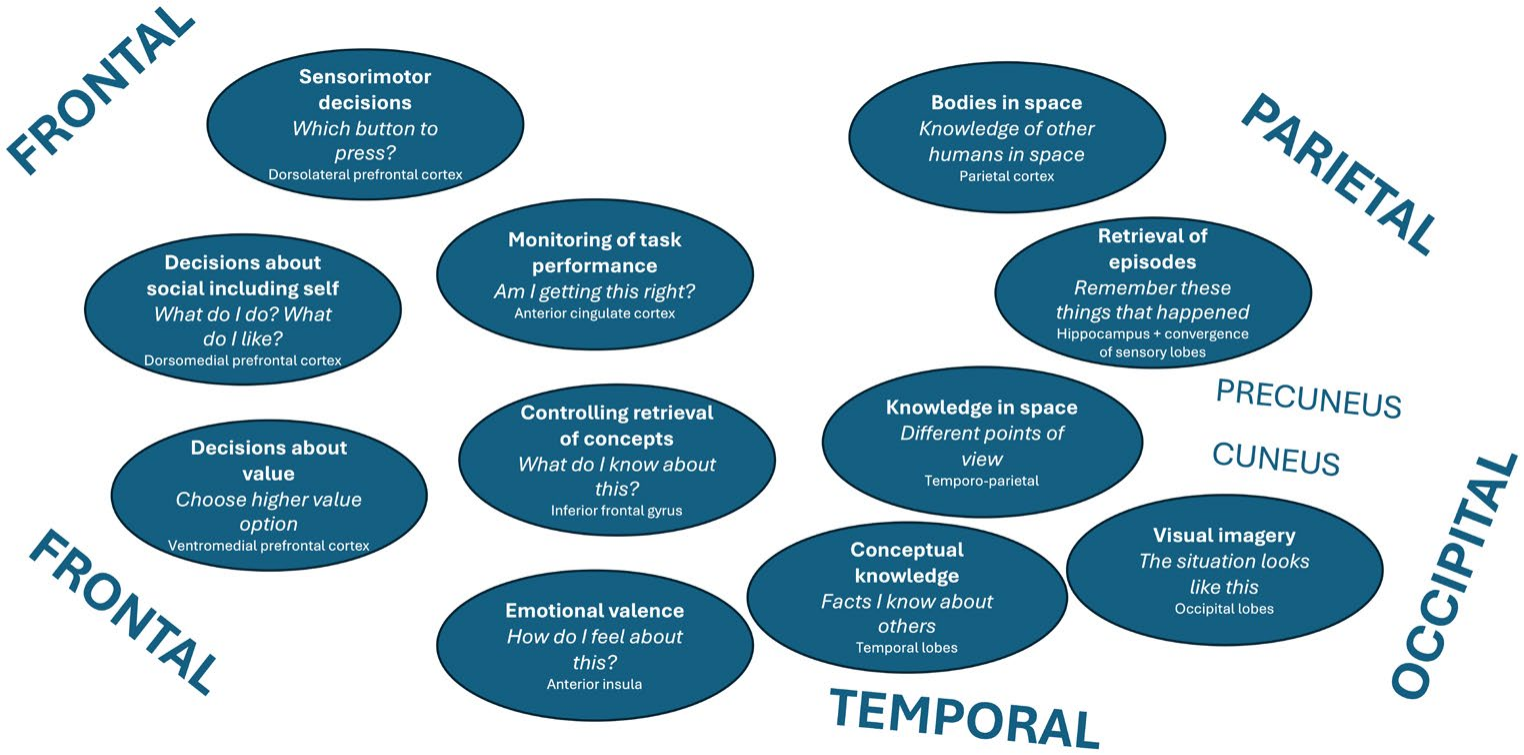
The functions that different brain regions contribute to in‐scanner task performance in Rodriguez Buritica et al.'s (2024) fMRI study (task: Does this adjective—e.g. ‘smart’—describe you/your friends/your teachers). Frontal areas are involved in controlling retrieval of memories and making decisions, including decisions based on task schemas about ‘self’; posterior sensory areas host concepts about self and others, their points of view and visual imagery, including of retrieved episodes and situations; and emotion processing areas add emotional value to concepts.

We saw earlier that educational neuroscience does not add value if it replaces useful psychological concepts with lists of brain areas. So, we need to make sense of the regions that Rodriguez Buritica et al. identify in terms of what they tell us about how the brain is carrying out evaluations. Figure 2 shows the main brain regions the authors identified and their likely functional contributions during the in‐scanner task. The front of the brain exerts control (such as activating knowledge and retrieving memories) and makes decisions (such as what button to press based on judgements of value or judgements about self) to achieve the task goal. The back of the brain is sensory, containing knowledge and concepts, including hosting imagery of retrieved memories. The sub‐cortical systems add emotional value to concepts. So, when individuals are asked to make judgements about traits in themselves or others, control processes become activated to retrieve the relevant knowledge, either based on memory episodes or conceptual knowledge, and then make value judgements by pressing a button. Rodriguez Buritica et al. report two main findings: the social decision‐making part of the front of the brain is more active in making decisions about self than others: this is because this area hosts task schema about self (it ensures you make decisions and choose behaviours consistent with who you are). Second, whether you are making a decision about friends or teachers modulates which regions become more active in the precuneus, a region of the somatosensory cortex adjacent to the visual cortex. This finding may reflect a differential balance of retrieval of episodic memories, use of imagery and access to concepts in making trait judgements (‘thinking’) about friends versus teachers.

The results of the imaging study highlight the challenge in this field. As Horvath and Donoghue (2016) urge, evidence from the neural level must be behaviourally translated before being useful to shape teaching practice. For this paper, although the neuroscience findings are intriguing in reflecting social, emotional and sensorimotor contributions to task performance, they do not at first blush make close contact with the behavioural data on academic self‐concept. They may instead provide insight into how children perform the in‐scanner task involving trait judgements rather than telling us about the formation and updating of academic self‐concepts and their role in academic achievement. We can distinguish, then, between the two perspectives that Rodriguez Buritica et al. offer. The behavioural data are educationally relevant, indicating pathways to help students improve academic self‐concept. The neuroscience data operate at the level of basic research, gradually exploring the link between self‐concepts, other‐person concepts and value judgements as instantiated in neural processing, here highlighting the possible importance of a visual imagery region.

### Menabò et al.

The third paper by Menabò et al., ‘What roles matter? An explorative study on bullying and cyberbullying by using the eye‐tracker’ (Menabò et al., 2023) also adopts the educational neuroscience approach of using more sensitive measures to reveal underlying processes, tapping neural mechanisms of visual attention. It takes on the serious issue of bullying, and here we see a study that seeks to provide stepping stones to intervention. In order to give children strategies about how to respond when they are involved in or witnessing bullying situations, it is important to understand how the children perceive and understand these situations. One can observe their behaviour in these situations and ask them to describe verbally how they understood them, but the authors argue that these qualitative measures are remote from how the children experience situations. Verbal reports, for example, may contain implicit prompts to produce conventional narrative structures with actors and patients, perpetrators and victims, thereby focusing on bullying only as a two‐person situation. In reality, bullying situations can also include those supporting the bully, defending the victim and bystanders. How does the child perceive these roles? For this, Menabò et al. turned to the more sensitive measure of eye‐tracking to tap the sorts of social information processing engaged in by the brain in experiencing these situations.

Fifty 10‐ to 11‐year‐olds were shown pictures representing vignettes of bullying situations, spanning physical bullying, verbal bullying, relational bullying and, notably, cyberbullying. Their gaze patterns across bully, victim, pro‐bully, defender and bystander were measured using indices such as fixation count, visit count and total fixation duration. These implicit measures were compared to verbal reports generated by children of the same vignettes. As expected, those verbal reports focused predominantly on the bully and victim. Yet, eye‐tracking measures showed the children were also aware of bystanders—what other people who witnessed the event were doing. Particular attention was paid to the defender in physical bullying and cyberbullying.

In contrast to the previous papers, the Menabò et al. article is more directly concerned with building an evidence base to guide intervention to reduce the impact of bullying on children's socioemotional development. Educational neuroscience measures such as eye‐tracking are used to better understand children's perception of the different roles involved in bullying and cyberbullying, and so inform interventions such as group discussions and role‐playing activities, which may, for example, encourage bystanders to intervene or to alert teachers. The effectiveness of such interventions could, in turn, be evaluated by these more sensitive measures—do they change the way children perceive bullying, and does the changed perception feed through to altered behaviours? These are, of course, questions for the next stage of this research—developing and evaluating interventions.

### Janssen and van Atteveldt

In the final paper of the special issue, Janssen and van Atteveldt's ‘Explore your brain: A randomized controlled trial into the effectiveness of a growth mindset intervention with psychosocial and psychophysiological components’ (Janssen & van Atteveldt, 2022), we see a study that has arrived at intervention. This means that issues of evaluation come to the fore. Key questions in evaluation revolve around the selection of control groups, randomization of participants to condition, the selection and timing of pre‐, post‐ and follow‐up measures, decisions about which outcomes to assess and where changes are expected, a focus on what effect size would be deemed worthwhile, and pragmatically, how much the intervention costs for the effect size it yields—many issues which are still the subject of debate in educational neuroscience (see, e.g. Thomas et al., 2024; Bowen et al., 2024).

The theory here is as follows. We are in the domain of meta‐cognition. The contention is that your ‘mindset’—your beliefs and expectations about how you think and learn—can affect your academic progress and your well‐being. If you know something about how your brain works, specifically that it is continually plastic and that you are in control of your academic progress by virtue of the effort you put in on the one hand and how you respond to setbacks on the other, then you will do better academically. By contrast, if you think that you do not have control over your abilities, that they are somehow fixed, this may impede academic progress because failure may be interpreted as inevitable rather than a spur to further effort. And this may indeed be one of the limiting factors for academic progress in children from low socioeconomic status (SES) backgrounds. A meta‐cognitive intervention is proposed that targets mindsets to shift beliefs from fixed (no control) to growth (control) by providing information about how the brain works.

The first three papers took the indirect pathway between neuroscience and education that proceeds via psychology. They were interested in using an understanding of neural mechanisms and neuroscience methods to improve psychological theories of reading development, academic self‐concept and comprehension of bullying situations respectively. In this fourth paper, we see the use of the indirect route between neuroscience and education that proceeds via meta‐cognition, where neuroscience can influence educational outcomes via self‐knowledge of how one's own brain works.

The novel idea in this study is that knowledge about how the brain works may not in itself be enough to convince students. The authors add an experiential element to their intervention that allows students to witness their ability to change brain function using a neurofeedback paradigm based on electrophysiology, inspired by the researchers' experience of using mobile brain‐imaging technology in the classroom (Janssen et al., 2021). When students concentrate, they can alter the electrical activity produced by mass neural firings, as measured by electrodes on the scalp. This additional element should, it is proposed, boost the effect of the meta‐cognitive intervention.

Several aspects of this study impress, including its scale: 439 participants (20 high‐school classes) randomly allocated to the two conditions; the duration of the intervention (four × 50‐min lessons); the resources (8 undergraduates were required to give the lessons); the uptake (95% of students attended 3 or 4 lessons) reflecting enthusiasm among participants; the rigour of the evaluation design (a parallel cluster randomized controlled trial following CONSORT‐SPI 2018 guidelines); the use of an active control group against which an intervention effect was assessed: the control group received lessons on how the brain works but *without* aspirational ‘growth’ messaging and the experiential neurofeedback component; and the range of outcome measures (mindset itself, overall grade point average, math grade, and the well‐being measure of school burnout) measured before, immediately after, and 1 year following the intervention.

What did Janssen and van Atteveldt find? Over the 1‐year period of the study, grade point average dropped for both intervention and control groups, and school burnout symptoms increased. If we combine this with evidence from Rodriguez Buritica et al. (2024) that student academic self‐concept also declines in early adolescence, these findings show that being a teenager in education in Western Europe is challenging. Concerning the intervention, it increased the number of students who indicated that they now held a ‘growth mindset’ (the authors added an extra level of granularity by distinguishing competitive vs. non‐competitive versions). Crucially, grade point average declined less in the intervention group, with a small effect size of *d = *.22, driven mainly by preventing the decline in math grades (*d = *.36). The authors replicated the finding that students from lower SES family backgrounds were more likely to have fixed mindsets (according to the self‐rating questionnaire), but the study did not replicate the finding that lower‐SES students were more likely to benefit from growth mindset interventions (Sarrasin et al., 2018; Sisk et al., 2018), nor that such interventions would reduce symptoms of school burnout (Kim, 2020).

The Janssen and van Atteveldt study represents the applied end of the research continuum. This means that theory must be turned into details of an educational intervention involving key decisions about implementation and evaluation. The central idea of an experiential element to a meta‐cognitive intervention, at least as presented in the paper, is plausible, but no substantial theory is offered to support it. Nevertheless, if this is a punt, it seems to pay off, with robust improvements in math achievement a year after the intervention ceased. There are some limitations to the study. The neural element of the intervention, the experiential neurofeedback, does not have any obvious link to mechanisms of learning. It might even be viewed as a placebo, exhorting participants to believe the intervention will work. In the main, the intervention improved mathematics achievement, and the authors admit that the intervention had more mathematics content than the control condition since it emphasized the growth mindset more with math examples than other subjects. The choice of the control group meant that the contributions of the growth mindset component and experiential neurofeedback element to the intervention were conflated. Some effects found in other research on growth mindset were not replicated, but this reminds us that in the context of evaluation, one study is never enough. Any one study may be subject to specific issues of context, sampling or implementation of the intervention. So here, we need to rely on the tools of meta‐science, such as systematic reviews and meta‐analyses, to build a robust evidence base. Lastly, the paper illustrates ongoing debates about effect sizes in educational interventions. Janssen and van Atteveldt maintain that an effect size of *d* = .36 is relatively large for the meta‐analytic average of *d* = .08 for growth mindset interventions (Sisk et al., 2018) but acknowledge that their intervention also had relatively high costs and intensity. Even when interventions are shown to be effective, to be implemented in schools, a key question is whether the effect size of the benefit is worth the resources invested. This is why the UK Educational Endowment Foundation has marshalled its evidence base to assess not just the effect size of various educational techniques and the strength of the evidence base supporting them but also their respective costs, because the combination is required to support schools in making decisions about whether to adopt the techniques (Coldwell et al., 2017).

## CONCLUSION

Together, the four papers in this special issue take us from basic science to applied intervention. They show us the different ways that neuroscience may inform key issues in education and illustrate the range of methods that educational neuroscience researchers bring to bear.

In this commentary, we have argued that educational neuroscience (mind, brain and education; neuroeducation) is inherently translational. We have argued that a focus on the brain matters for teachers because it increases understanding of how learning works and the factors that influence learning outcomes and student well‐being, without being reductionist. A neuroscience perspective encourages a more holistic and developmental view of learning than a narrow cognitive (memory) oriented approach.

As an inherently translational field that relies on dialogues between researchers and practitioners, we argued that it is crucial to understand how teachers view educational neuroscience as informing their pedagogy and how, in reality, that can be fitted into their professional lives—whether this is in the form of CPD or ideally in teacher training, where we considered the current UK government's initial teacher training and early career frameworks which lay out what the government thinks that teachers should know about learning.

We conclude that knowledge of how the brain works in educational contexts needs to be turned into a form that is useful to teachers in the classroom. However, many forms of study will converge to produce this desirable outcome.

## AUTHOR CONTRIBUTIONS

**Michael S. C. Thomas:** conceptualization (equal); writing – original draft (equal); writing – review and editing (equal). **Yasin Arlsan:** conceptualization (equal); writing – original draft (equal); formal analysis (lead); writing – review and editing (equal).

## CONFLICT OF INTEREST STATEMENT

The authors declare no conflicts of interest.

## Data Availability

Data available from the authors on request.
